# Sensory Phenotypes in Autism Spectrum Disorder Associated with Distinct Patterns of Social Communication, Repetitive and Restrictive Behaviors or Interests, and Comorbidities: A State-of-the-Art Review

**DOI:** 10.3390/brainsci16010053

**Published:** 2025-12-30

**Authors:** Carla Consoli, Laura Turriziani, Marta Antoci, Marianna Lo Monaco, Graziana Ceraolo, Giulia Spoto, Antonio Gennaro Nicotera, Gabriella Di Rosa

**Affiliations:** 1 Unit of Child Neurology and Psychiatry, Department of Human Pathology of the Adult and Developmental Age “Gaetano Barresi”, University of Messina, 98125 Messina, Italy; carlaconsoli@hotmail.com (C.C.); marta.antoci94@gmail.com (M.A.); marianna.lomonaco2@gmail.com (M.L.M.); graziana.c23@hotmail.it (G.C.); 2 IRCCS-Centro Neurolesi Bonino-Pulejo, S.S. 113, Via Palermo, C. da Casazza, 98124 Messina, Italy; 3 Unit of Child Neurology and Psychiatry, Department of Biomedical Sciences, Dental Sciences & Morphofunctional Imaging, University of Messina, 98125 Messina, Italy; giulia.spoto27@gmail.com (G.S.); gdirosa@unime.it (G.D.R.); 4 Unit of Child Neurology and Psychiatry, Maternal-Infantile Department, University of Messina, 98125 Messina, Italy; antonionicotera@ymail.com

**Keywords:** autism spectrum disorders, sensory processing, sensory phenotypes, sensory integration therapy

## Abstract

Sensory processing differences, reported in up to 97% of individuals with autism spectrum disorder (ASD), are increasingly recognized as a defining feature of the condition, shaping perception, cognition, and adaptive behavior. Atypical sensory responsivity, ranging from hyper- to hypo-reactivity and sensory seeking, emerges early in development and contributes to the clinical and neurobiological heterogeneity of autism. Alterations in neural connectivity, the balance of excitation and inhibition, and multisensory integration are thought to underlie these sensory profiles, influencing emotional regulation, attention, and social interaction. Sensory features also interact with co-occurring conditions such as anxiety, attention deficit hyperactivity disorder, and sleep and feeding difficulties, thereby shaping developmental trajectories and influencing adaptive behavior. Clinically, these sensory dysfunctions have a significant impact on daily participation and quality of life, extending their effects to family functioning. Understanding individual sensory phenotypes is therefore essential for accurate assessment and personalized intervention. Current therapeutic approaches include Sensory Integration Therapy, Sensory-Based Interventions, Sequential Oral Sensory Approach, and structured physical activity programs, often complemented by behavioral and mindfulness-based techniques. Emerging neuroplasticity-oriented methods for targeted modulation of sensory processing networks include neurofeedback and non-invasive brain stimulation. Overall, current evidence highlights the central role of sensory processing in ASD and underscores the need for multidisciplinary, individualized approaches to optimize developmental trajectories and enhance adaptive functioning. This review provides an updated synthesis of sensory processing in ASD, integrating neurobiological, developmental, and clinical evidence to highlight established knowledge, unresolved questions, and priorities for future research.

## 1. Introduction

Autism Spectrum Disorder (ASD) is a neurodevelopmental condition characterized by challenges in social interaction, communication, and repetitive behaviors [1]. Sensory differences have been described since the earliest clinical description of autism in the 1940s, but only with the release of the fifth edition of the Diagnostic and Statistical Manual of Mental Disorders (DSM-5) in 2013 were they formally incorporated into diagnostic criteria and considered as core features of ASD [1].

Studies estimate that 60% to 97% of individuals with ASD exhibit atypical sensory processing reflecting methodological heterogeneity across assessment tools, age groups, and diagnostic thresholds [2,3,4,5,6]. Sensory processing is the ability to receive incoming sensory information from the surrounding environment and generate behavioral responses accordingly. This adaptive interaction is fundamentally shaped by two interrelated components: neurological thresholds and self-regulatory behavior. The former defines the level of sensory input necessary for detection and recognition, whereas the latter represents the individual’s behavioral responsiveness to such sensory stimuli [7]. In this context, sensory profile refers to patterns of observable behavioral responses to sensory input across modalities, typically assessed through standardized caregiver-report questionnaires such as the Sensory Profile or Short Sensory Profile (see Table 1) [8].

Typical sensory profiles include hyper-reactivity (e.g., aversion to sound or light), hypo-reactivity (e.g., indifference to pain or name-calling), sensory seeking (e.g., fascination with textures or lights), and enhanced perception (e.g., heightened attention to detail). These dimensions often coexist and vary in intensity across sensory modalities such as visual, auditory, tactile, olfactory, gustatory, proprioceptive, and vestibular domains [9].

Although some sensory abnormalities can be highly distressing and negatively impact quality of life, others may be experienced as pleasurable, soothing, or even rewarding by individuals with ASD. Many adults with ASD intentionally seek out enjoyable and calming sensory experiences—such as listening to favorite music, touching certain textures like cold, smooth surfaces, or smelling preferred scents such as food aromas, perfumes, or essential oils—highlighting that sensory seeking may serve adaptive and self-regulatory functions [10]. Recognizing this variability is essential, as not all sensory differences necessarily constitute deficits. In certain cases, they may reflect distinctive perceptual strengths or heightened sensitivities that prove advantageous in specific contexts. For example, some individuals with ASD demonstrate enhanced music pitch discrimination, absolute pitch, and superior spatial awareness, which may be regarded as assets contributing to creativity and attention to detail [11,12,13].

Multisensory processing is crucial for constructing a coherent perceptual and cognitive representation of reality [14]. Emerging evidence links sensory phenotypes to atypical neural mechanisms, including altered thalamocortical connectivity, cortical microcircuit mechanisms, disrupted cortical excitatory–inhibitory (E–I) balance, impaired temporal and neuromodulatory mechanisms [15,16,17,18]. Sensory phenotype is a multidimensional construct that integrates patterns of sensory responsivity, modulation, and neural processing across sensory modalities, potentially reflecting underlying neurobiological mechanisms (see Table 1) [19,20]. Individuals with ASD often show altered integration of audiovisual stimuli (e.g., speech perception) [17] and prolonged temporal binding windows (TBW), reflecting less precise integration of sensory information across time [18]. Hypoactivity of the medial prefrontal cortex and reduced long-range connectivity may impair top-down modulation, increasing focus on low-level perceptual details. Reduced synchrony between cross-modal neural systems has been reported in children with ASD, which may improve with age [14,21]. These impairments likely contribute to broader difficulties in social cognition, communication, and adaptive behavior [22,23].

Sensory features may thus mediate the relationship between early neurodevelopment alterations and the expression of autistic symptoms. Although the function of sensory behaviors in young children is still largely unexplored, specific sensory behaviors emerging early in development may predict later autistic traits. Bussu et al. [7] showed that distinct sensory behaviors in infancy are shaped by separable etiological influences and independently predict autistic traits in toddlerhood. Sensory seeking may be particularly heritable, and it is considered an early emerging trait linked to ASD risk. On the contrary, hyper-reactivity, being more environmentally influenced, may be more responsive to early interventions [7].

Increasing evidence indicates that sensory and social processing are closely linked. In fact, Fattal et al. [24] showed that disruptions in interpersonal synchronization across development are critical to understanding social dysfunction in ASD. Social functioning relies on the dynamic coordination of explicit and implicit behavioral and attentional processes, collectively termed “social cognition”. Early sensory dysregulation may disrupt attention to social cues, joint attention, and imitation [24,25]. Impaired interpersonal synchronization, rooted in atypical sensory input, contributes to social difficulties [24,26].

Sensory abnormalities also affect internalizing (e.g., anxiety) and externalizing behaviors (e.g., aggression). A recent meta-analysis found that hyper-reactivity strongly predicted both internalizing and externalizing problems in autism, while atypical smell/taste processing showed a weaker association, and sensation seeking relates primarily to externalizing problems [27]. Distinct sensory phenotypes showed differential associations with ASD symptomatology: hyper-reactivity with anxiety, social withdrawal, and defensiveness [28]; hypo-reactivity with joint attention deficits and reduced social responsivity; and sensory seeking with stereotyped behaviors and executive dysfunction [29,30,31]. These findings indicate that sensory reactivity may play a developmental role in the manifestation and maintenance of core ASD features rather than constituting a secondary characteristic [32].

Moreover, a bidirectional relationship exists between sensory processing, cognitive function, and sleep quality: sleep disturbances can exacerbate sensory dysfunction, creating a cycle that affects daily functioning and learning [33,34].

Sensory processing differences are detectable in infants well before a formal ASD diagnosis. Early neurological assessment may help identify altered neurodevelopmental trajectories in high-risk populations such as preterm children, in whom visual and social skills appear to be the most affected [35,36,37,38].

Moreover, genetic analyses have identified candidate genes (e.g., *SHANK3*, *SCN2A*, *CNTNAP2*, *MECP2*) implicated in multisensory processing differences, suggesting shared heritability with core ASD traits [17,39,40,41]. Notably, extensive literature has linked various genetic factors to ASD, supporting the hypothesis that specific alterations may act as primary or contributing mechanisms in its neurodevelopmental pathogenesis [42,43,44,45,46,47,48]. In addition, immune and inflammatory dysregulation have been proposed to contribute to the pathogenesis of autism through neuroinflammation, influencing both the course and the severity of the symptomatology [49,50,51,52].

Overall, current evidence points to early sensory processing differences as potential biomarkers of ASD risk and developmental outcomes. To guide the reader through this complex literature, this review is structured to reflect the multilevel nature of sensory processing in ASD. We first examined the neurobiological mechanisms proposed to underlie atypical sensory processing. Building on this foundation, we then described how sensory processing differences manifest across specific sensory modalities and how these patterns interact with development and co-occurring conditions. The final sections discuss sensory-focused assessment and intervention approaches, including their mechanisms, applicability, and supporting evidence. Taken together, this review aims to provide a comprehensive and updated overview of sensory processing differences in individuals with ASD, integrating neurobiological, behavioral, and clinical perspectives to clarify their relevance for early identification and intervention, and to highlight directions for future research.

## 2. Methods: Literature Selection Strategy

**Figure 1:**
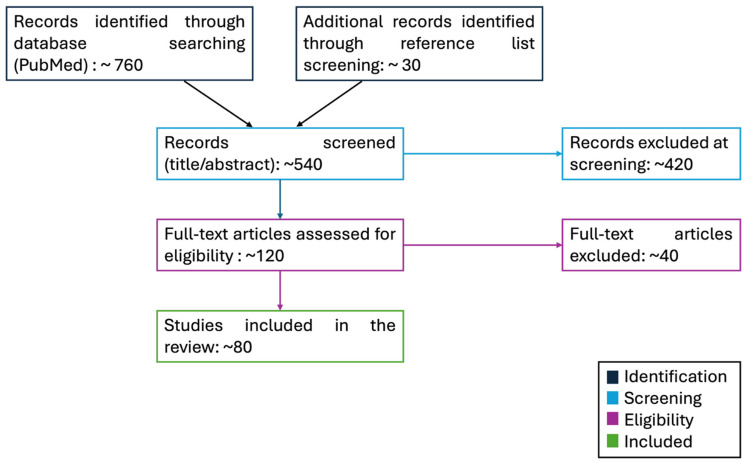
Flow diagram: Overview of the literature search and selection process used to inform this narrative review. Records were identified from database searches and reference list screening, followed by title/abstract screening and full-text assessment. Numbers are reported as approximate values to enhance transparency without implying a systematic review.

This narrative review was informed by a structured literature search conducted in PubMed using combinations of keywords related to autism and sensory processing (including *autism*, *ASD*, *sensory processing*, *sensory reactivity*, and *sensory phenotypes*). The search covered studies published between 1990 and October 2025, and the last search was performed in October 2025; earlier seminal and historical works were included when relevant through reference list screening. Titles and abstracts were screened for relevance, and full texts were examined when necessary. Studies were considered eligible if they addressed sensory processing or sensory reactivity in individuals with ASD and provided behavioral, neurobiological, physiological, or intervention-related data directly relevant to sensory phenotypes; studies focusing on non-ASD populations or lacking direct relevance to autism-related sensory features were excluded. Additional relevant articles were identified through manual screening of reference lists of key reviews. Given the narrative scope of the article, no formal meta-analysis or risk-of-bias assessment was performed. The literature search and selection process are summarized in a compact flow diagram (see Figure 1), with article counts reported as approximate values. This approach was adopted to improve transparency while preserving the narrative scope of the review.

## 3. Neurobiological Mechanisms Underlying Atypical Sensory Processing in ASD

Sensory abnormalities in ASD arise from atypical processing mechanisms rooted in altered neurodevelopment, rather than from acquired sensory dysfunctions as observed in other neurologic and psychiatric conditions [3,53,54,55,56]. The integration of sensory information is essential for building coherent perceptual experiences and supporting cognitive functions. This process relies on specialized neural circuits and regions, extending from the spinal cord and brainstem to the cerebral cortex, specifically dedicated to combining inputs from different sensory modalities [14]. Circuit dysfunction within these multisensory integration pathways may contribute to the atypical sensory processing frequently observed in autism [15].

Atypical sensory processing in ASD can be conceptualized across four interacting domains. First, cortical microcircuit alterations—including E–I imbalance, parvalbumin-interneuron dysfunction, and reduced gating and habituation—disrupt neural gain control and temporal precision. Second, temporal and multisensory integration deficits, such as broadened TBW and atypical thalamocortical or long-range connectivity, impair the coordination of inputs across sensory modalities. Third, sensory coding and computational anomalies—including degraded sensory representations and altered weighting of priors—contribute to heightened reliance on raw sensory input and reduced contextual modulation. Finally, neuromodulatory pathways may further modulate sensory responsivity and plasticity.

### 3.1. Cortical Microcircuit Mechanisms

Several theories have been proposed to explain sensory circuit dysfunction in ASD, including alterations in the E–I balance, leading to altered network excitability rather than uniformly increased hyperexcitability. This imbalance is thought to arise from altered neurotransmission and synaptic functioning in regions such as the neocortex, cerebellum, hippocampus, and amygdala [15]. Contributing factors include reduced levels of inhibitory neurotransmitters (e.g., GABA), deficits in synaptic proteins (e.g., neuroligins), and possible immune-related changes (e.g., altered cytokine profiles) [9,57]. Together, these alterations can affect cortical microcircuits, with consequences for information processing, behavior regulation, and functions such as motor control and reward processing [58]. A systematic review of transcranial magnetic stimulation (TMS) studies found that short interval intracortical inhibition is likely reduced in individuals with ASD compared with controls, a pattern consistent with GABAergic dysfunction. This reduction in cortical inhibition represents a plausible neurophysiological mechanism contributing to sensory hyper-reactivity in ASD [59]. Consistent findings emerged from multimodal approaches: Bernardino et al. [60], combining magnetic resonance spectroscopy (MRS) and TMS, reported an E–I imbalance characterized by atypical GABA_A_ receptor-mediated inhibitory dynamics and variations in glutamate levels, suggesting a functional disruption of inhibitory circuits even in the absence of marked reductions in global GABA concentrations [60]. Independent MRS studies further supported this interpretation. In both children and adults with ASD, reduced GABA concentrations have been reported in perisylvian and sensory cortical regions, associated with linguistic, perceptual, and sensory processing difficulties [61]. Likewise, neurophysiological reviews reported altered electroencephalography (EEG)/magnetoencephalography (MEG) sensory responses—including atypical gamma-band modulation that depends critically on GABAergic interneurons function—which have been linked with sensory hyper-reactivity [20,62].

However, because many individuals with ASD also show hypo-reactivity and sensory seeking, E–I imbalance and hyperexcitability alone cannot fully account for the diverse sensory phenotypes in autism [15].

Additional mechanisms have therefore been proposed to further explain sensory processing differences in ASD. One prominent hypothesis involves the hypofunction of parvalbumin-expressing interneurons, which represent almost 40% of cortical interneurons and provide strong perisomatic inhibition to pyramidal neurons [15,63]. Post-mortem brains and mouse studies have shown reduced expression, number, and function of parvalbumin cells in ASD, which can disrupt neuronal precision, increase pyramidal firing, disturb gamma synchronization, and degrade spike timing [63,64,65]. Parvalbumin interneurons hypofunction also contributes to abnormalities in gamma synchrony [66] and may also impair critical period plasticity, resulting in abnormal or immature neural circuit development [67].

Another mechanism implicated in ASD is the altered sensory gating, referring to the brain’s ability to suppress responses to repetitive or irrelevant stimuli, thereby preventing sensory overload and maintaining attentional focus [68].

Neurophysiological data further support atypical sensory processing in ASD, with impaired suppression of event-related potentials such as P50, N100, and P200 contributing to distractibility and reduced filtering of environmental stimuli [9,69,70].

Similarly, impaired habituation, defined as a reduced ability to diminish neural or behavioral responses to repeated stimuli, has been documented across sensory modalities in individuals with ASD [71]. Longitudinal electrophysiological studies showed reduced auditory habituation in infants at elevated likelihood for ASD, whereas eye-tracking studies revealed atypical attentional disengagement and reduced adaptation to visual and multimodal events [72,73]. A reduced capacity to adapt to or filter out unchanging environmental input may contribute to heightened sensitivity and the persistence of stereotyped behaviors in autism [72,74].

In summary, although several mechanisms have been proposed, the most consistently supported mechanism remains the alteration of the E–I balance, particularly involving GABAergic dysfunction, as shown across TMS, MRS, and EEG/MEG findings [20,59,60,61]. Other mechanisms, such as parvalbumin-interneuron hypofunction or atypical sensory gating and habituation, remain biologically plausible but are more weakly established in humans, relying heavily on animal models or heterogeneous electrophysiological paradigms [66,72]. Available data also suggest partial differentiation at the phenotypic level: reduced GABAergic inhibition and gamma-band abnormalities have been most consistently linked to sensory hyper-reactivity, attenuated early evoked responses appear more compatible with hypo-reactivity profiles, and reduced habituation—observable from infancy—has been associated with later sensory seeking tendencies [20,72,75]. The interpretation of these patterns remains limited by small and heterogeneous cohorts, developmental influences, and the variable reliability of electrophysiological measures, emphasizing the need for larger and methodologically standardized studies to delineate the relative contribution of each mechanism.

### 3.2. Temporal and Multisensory Integration Mechanisms

Deficits in temporal and multisensory integration are also well-documented neurobiological alterations in ASD and can impair the ability to recognize and respond to stimuli, thereby affecting various social and cognitive functions [9]. Atypical multisensory processing may lead to difficulties in integrating information from different sensory channels, resulting in fragmented or delayed perceptual experiences that negatively impact social communication [76]. Literature data suggested that the dysfunction might be correlated to the size of the TBW, a critical time within which paired stimuli are highly likely to be perceptually bound [77]. Most studies reported a broader TBW in ASD, which can reduce the precision of temporal integration and lead to atypical perception of illusions such as the sound-induced flash effect, a commonly used measure of audio–visual integration [77,78,79].

Evidence from Foss-Feig et al. [80] and Stevenson et al. [81], together with meta-analytic syntheses, confirmed that an enlarged TBW was a robust and reproducible feature of ASD across developmental stages [80,81,82]. Recent multimodal studies further indicated that multisensory differences can emerge at early electrophysiological latencies: Dwyer et al. [83] showed that while adolescents with ASD demonstrated preserved behavioral multisensory facilitation, their audiovisual event-related potential interactions were attenuated and delayed relative to controls, suggesting reduced efficiency of early cross-modal binding processes [83]. Complementary neuroimaging findings further reported altered thalamocortical coupling and reduced long-range functional connectivity, among auditory, visual, and parietal regions [16,84], providing a plausible circuit-level basis for reduced integration efficiency. These connectivity patterns fit within a broader literature describing a combination of both local overconnectivity and long-range underconnectivity in ASD, a configuration that may contribute to uneven or fragmented perceptual experiences [85,86]. Despite these convergent findings, substantial methodological heterogeneity—ranging from task variability to small samples and the sensitivity of EEG, MEG, or neuroimaging to motion and analytic noise—still limits comparability across studies and underscores the need for harmonized, longitudinal approaches [87].

### 3.3. Sensory Coding and Computational Mechanisms

Alongside circuit-level alterations, recent research has identified two complementary mechanisms that may help explain sensory processing differences in autism: degraded sensory coding and atypical predictive coding. Degraded coding refers to alterations in the neural encoding of sensory information that reduce the quantity or precision of information available for accurate detection and discrimination [15]. This alteration can lead to atypical sensory experiences and contribute to behavioral traits such as sensory seeking, sensory aversion, or insistence on sameness. Evidence from various ASD models suggests that such coding impairment results in sensory representations that are noisy, unstable, or poorly defined [15,88,89].

In parallel, predictive coding theories suggest that individuals with ASD may tend to assign less emphasis to prior expectations, which could make perception more dependent on raw sensory input and less influenced by context. This may lead to heightened sensitivity to irrelevant details and inflexibility in perceptual adaptation [90,91].

Supporting this interpretation, Lawson et al. [92] showed that adults with ASD overestimate environmental volatility, consistent with reduced confidence in predictions. In a theoretical review, van de Cruys et al. [93] synthesized existing findings to argue for atypically high and inflexible precision of prediction errors in ASD. These computational profiles are consistent with heightened sensitivity to small changes, difficulties filtering irrelevant input, and a strong reliance on sameness [90,92,93]. However, compared with inhibitory imbalance or multisensory integration, the empirical support for these computational mechanisms remains more heterogeneous, relying on fewer human studies and on theoretical models that require further validation through longitudinal and multimodal approaches.

### 3.4. Neuromodulatory Mechanisms

Beyond circuit- and computation-level accounts, neuromodulatory and immune–inflammatory pathways have also been implicated in sensory processing differences in ASD. However, the evidence supporting these mechanisms remains more preliminary and heterogeneous than that for inhibitory imbalance or multisensory integration. Among neuromodulatory systems, emerging evidence also highlights the critical role of the oxytocin (OXT) system in modulating sensory processing [94,95]. OXT, a nonapeptide mainly synthesized in the supraoptic and paraventricular nuclei of the hypothalamus, acts through receptors that are widely expressed across sensory-related brain regions and circuits [96]. OXT plays a crucial role in social bonding and is released in response to affective tactile stimuli, such as touch, warmth, and social odors [97]. In individuals with ASD, abnormalities in the OXT system have been documented at multiple levels. Modahl et al. [98] found significantly reduced plasma OXT levels in children with ASD compared to neurotypical controls, suggesting a diminished systemic oxytocinergic tone. Converging evidence showed that both OXT and arginine vasopressin modulate human social and affective behavior, including tactile and olfactory processing, providing a mechanistic link to sensory responsivity [99]. Genetic data further support this pathway: Campbell et al. [100] identified multiple OXT receptor gene variants associated with core ASD phenotype domains, including social communication and repetitive behaviors, which may indirectly intersect with sensory modulation. Camerino recently describes how disrupted OXT signaling may impair thermoregulation, potentially contributing to atypical sensory experiences in autism, although evidence remains preliminary and does not yet allow a precise mapping between OXT-related mechanisms and specific sensory phenotypes (e.g., hyper-reactivity vs. hypo-reactivity) [94,95].

Overall, atypical sensory processing in ASD reflects the complex interaction of multiple neurobiological mechanisms, collectively shaping the heterogeneous sensory and behavioral profiles observed across individuals with ASD. These neurobiological mechanisms do not exert uniform effects across all modalities, but contribute to distinct patterns of tactile, visual, auditory, olfactory, gustative, and interoceptive processing. Therefore, to translate neural mechanisms into observable behaviors, the following sections examined domain-specific sensory phenotypes in autism.

## 4. Domain-Specific Sensory Processing

Sensory processing differences have become a central focus of clinical assessment and intervention in ASD, as reflected in their inclusion in the DSM-5 diagnostic criteria [9].

These alterations rarely occur within a single modality. Instead, they typically involve multiple sensory systems, including touch, vision, audition, taste, smell, and interoception, highlighting the pervasive and heterogeneous nature of sensory dysfunction in ASD [14,101].

However, for the sake of clarity and conceptual organization, we have nonetheless discussed sensory processing alterations according to the predominant sensory modality affected, describing their behavioral manifestations, underlying mechanisms, and implications for intervention. Understanding how these differences manifest across distinct sensory domains provides crucial insight into the perceptual and behavioral variability observed in individuals with ASD.

### 4.1. Tactile Processing in ASD

Touch is one of the fundamental sensory channels in human development, playing a crucial role in communication, social bonding, and environmental exploration, and supports the maturation of neocortical areas responsible for processing sensory input, such as the somatosensory cortex [102].

Atypical responses to tactile stimuli have been reported in approximately 60% of individuals with ASD who exhibit sensory processing differences that typically emerge in infancy (often before the age of 3) [4,103]. Both children and adults with ASD may show altered tactile reactivity to vibration, thermal pain, and light touch, affecting both smooth and hairy skin [19,29,104,105]. Interestingly, a neurophysiological study performed in Fragile X syndrome—a major genetic model of ASD—showed increased amplitudes of middle-latency somatosensory evoked potentials. These findings suggested a cortical dysfunction, outlined by hyperexcitability and impaired sensory gating [106]. Similarly, enhanced early somatosensory evoked potential peak were found in a population of young ASD children in the right hemisphere response [107]. These data may underlie the atypical tactile reactivity seen in ASD, linking both conditions through shared mechanisms of disrupted E–I balance.

However, these findings should be interpreted with caution, as both previously cited studies were based on very small samples (one including 10 patients and 9 controls, and the other involving 24 children without a control group). Such limited sample sizes and methodological constraints restrict the generalizability of the findings and make it difficult to determine whether the observed somatosensory alterations reflect broader ASD-related mechanisms or individual variability.

Difficulties in tactile processing, along with reduced tolerance for affective touch, can limit the ability to form secure attachment relationships, interact effectively with their surroundings, and receive nurturing contact, thereby hindering healthy development [40,108]. Moreover, they may contribute to social withdrawal and increased levels of anxiety, stress, and depression, which are frequently observed in the ASD population [97].

Evidence from Voos et al. [109] demonstrated through functional magnetic resonance imaging (fMRI) that affective tactile stimulation selectively engages a social brain network, including superior temporal sulcus, medial pre-frontal cortex, insula, orbito-frontal cortex, and amygdala, while higher autistic traits are associated with attenuated neural responses to C-tactile–targeted touch [109]. However, the small neurotypical-only sample and the absence of a clinical ASD group limit the generalizability of these results and make it unclear whether the reduced responsivity reflects ASD-related mechanisms or normal variability. Moreover, the study design does not distinguish sensory-driven responses from top-down influences such as attention, expectation, or mental imagery. As noted by the authors, occipital activation during slow touch may therefore reflect imagery-related processes rather than primary sensory processing [109].

Atypical tactile responsiveness in ASD generally manifests in three main patterns: heightened reactivity (hyper-reactivity), reduced reactivity (hypo-reactivity), and unusual tactile-seeking behaviors, all of which may be related to core ASD symptoms [29,40]. Tactile hyper-reactivity may manifest as distress during grooming, discomfort with clothing or footwear, avoidance of going barefoot, or difficulty tolerating close physical proximity [110]. This sensory phenotype is specifically associated with inflexible and repetitive behaviors, repetitive verbalizations, atypically focused attention, and sleep disturbances (e.g., alterations in falling asleep and maintaining the sleep state) [111,112,113]. Additionally, tactile hypo-reactivity may present as reduced sensitivity to pain and temperature, disregard for physical messiness, and is associated with impaired social functioning, reduced nonverbal communication skills, and increased repetitive behaviors [29,110]. Higher levels of tactile-seeking responses correlate with more severe repetitive behaviors and social impairments. Patterns of tactile hypo-reactivity and tactile-seeking behaviors may, in part, be explained by dysfunctions in neural reward systems that reduce sensitivity and responsiveness to social and environmental cues. Consequently, individuals may pursue alternative, non-social sources of stimulation and reward [29].

These studies adopted a solid methodological approach, focusing on a single sensory modality and combining direct observational measures with caregiver reports to enhance ecological validity. However, these works typically rely on relatively small samples, lack objective instrumental assessments, and do not directly compare different sensory systems. This limits the ability to clarify the specific contribution of each modality to core ASD symptoms and to inform the development of more targeted and effective interventions [29,110,113].

Closely related to tactile sensory processing is pain perception, a universal “alert” mechanism that protects the body from chronic and repetitive injury [114]. Proper pain regulation is essential for adaptive functioning, including the development of social interaction skills and empathy, and has been implicated in the social difficulties observed in ASD [115]. The literature on pain perception in individuals with ASD is heterogeneous, with a predominance of reports describing decreased or absent pain reactivity. Atypical pain experiences documented in ASD include allodynia (intense pain in response to normally non-painful tactile stimuli), paradoxical heat sensation (gentle cooling perceived as burning or hot), and hypoesthesia (reduced sensitivity to pain) [114].

This variability is compounded by methodological limitations, including small or clinically unbalanced samples, heterogeneous assessment approaches, and limited use of objective physiological measures. Pain processing in ASD likely reflects the interaction of multiple levels—from nociceptive mechanisms to sensory modulation, emotional appraisal, and communicative behaviors—suggesting that atypical pain responses may reflect differences in awareness or expression rather than reduced nociception. Consequently, comprehensive, multimodal assessment strategies are needed to more accurately characterize pain in ASD and inform tailored clinical interventions.

Overall, the literature supports a robust association between atypical tactile processing and core behavioral features of ASD, whereas evidence based on neurophysiological and neuroimaging evidence remain preliminary. Progress in the field will require larger, well-controlled, developmentally informed studies that integrate objective instrumental measures with behavioral assessments and directly compare tactile processing across sensory modalities. Such approaches are essential to clarify causal mechanisms and to establish the developmental and clinical significance of these findings, ultimately informing the design of targeted, sensory-informed interventions.

### 4.2. Visual Function in ASD

Vision represents a primary channel for acquiring environmental information [116]. The ability to perceive facial expressions and gestures is frequently reduced in individuals with ASD, thereby impacting communication, behavior, and quality of life [117,118]. Atypical gaze behaviors further illustrate the heterogeneity of visual strategies in ASD. Children may use a “lateral gaze,” observing objects peripherally while the head is oriented away, possibly to regulate the influx of visual information and reduce central overload. These alterations are frequently associated with an enhanced focus on perceptual details at the expense of global integration, described as “seeing the trees, but not the forest” [119].

Robertson et al. [120] demonstrated that difficulties in global motion perception in ASD may arise from atypical early visual processing rather than deficits in higher-order integration. The study showed marked reductions in both behavioral performance and neural responses in the primary visual cortex and the middle temporal complex when motion stimuli are low in coherence or presented for short durations. Despite a solid combined behavioral–fMRI approach, the sample was small and consisted exclusively of male, and mechanistic interpretations lacked direct neurophysiological measures. Moreover, the focus on a single sensory modality limited generalization to broader sensory profiles in ASD.

Such atypical visual perception emerges early in development and represents a key marker for early diagnosis, as reduced or absent visual fixation can be observed since the first years of life in children who will develop ASD [117].

Broad sensory differences, including hyper- and hypo-reactivity, have also been described [121,122]. Hyper-reactivity is characterized by extreme focus on small details, avoidance of eye contact, and light aversion, whereas hypo-reactivity involves attraction to bright lights and moving objects, prolonged gazing at people, and reduced sensitivity to differences in visual input intensity in the luminance domain [121,123]. Individuals with hypo-reactivity often experience greater communication difficulties, such as challenges in recognizing inappropriate statements during conversation, and reduced awareness of visual information in their surroundings, for instance failing to notice when someone enters a room [123]. Visual hypo-reactivity is further associated with higher frequencies of repetitive and stereotyped behaviors [124], and difficulties in attention switching or persistent focus may underlie reduced responsiveness to visual stimuli [123].

Neurobiological mechanisms underlying atypical visual behaviors in ASD have been extensively investigated. Early hypotheses emphasized deficits in oculomotor inhibitory control; however, more recent evidence suggested that the primary issue lies in altered integration of sensory and corollary discharge signals into motor commands, rather than in reflexive suppression itself [125]. Dysfunction of the dorsal visual stream and disrupted connectivity within visual cortical areas have been reported, along with subcortical abnormalities in the superior colliculus, which is critical for saccade generation [126]. Reduced cerebellar vermis activation during saccadic movements has been implicated in slower gaze adaptation [127]. Such dysfunctions may impair the regulation of saccade frequency, limiting environmental exploration and learning [128]. Additionally, structural anomalies, including atypical neuronal migration in the anterior cingulate cortex, may contribute to social cognition and theory of mind deficits via connections with the frontal cortex and temporoparietal junction [126,129].

The cognitive process underlying visual search typically involves three main stages: feature identification (e.g., color), attentional orientation, and executing goal-oriented actions focused on specific stimulus characteristics [130].

Individuals with ASD often exhibit atypical visual behaviors, including persistent and purposeless exploration of the environment, reduced fixation on socially relevant stimuli, and difficulty disengaging or shifting attention [117,130,131]. One of the most evident manifestations of socially oriented visual–perceptual deficits is abnormal eye contact, which directly affects joint attention (e.g., the ability to coordinate attention with a social partner toward an object or event). Joint attention deficits are considered among the earliest and most prominent symptoms of ASD [128]. Particularly, children with ASD show altered visual patterns when initiating joint attention, but not when responding to it. Impairments related to joint attention include difficulties in disengaging from faces, scanning global scene, and anticipating object actions [73].

A strong reliance on central visual targets during fixation suggests that, in their absence, individuals with ASD have reduced ability to recruit alternative cues, limiting attentional flexibility [131]. Consequently, visuospatial disorientation, visual avoidance, and excessive visual exploration are frequently described [117,132]. These abnormalities often involve oculomotor control, attentional processes, and visual–motor integration, with cascading effects on learning and social development [128].

Atypical visual processing also extends to object and face recognition. Individuals with ASD often show reduced attention to human faces, atypical processing strategies, longer reaction times, and lower accuracy in facial identification [117,133]. These deficits limit rapid and accurate understanding of others’ expressions and emotions, thereby reducing social interactions. Evidence suggests that these impairments may be associated with decreased sensitivity to luminance and contrast differences, which are critical for object recognition and particularly salient in face perception [117,123].

Recent theoretical frameworks suggested that visual differences in ASD may reflect not a uniform deficit, but rather divergent perceptual strategies characterized by enhanced processing of local elements and reduced efficiency in global integration. However, despite an expanding body of work on visual function, the current literature remains highly heterogeneous. Most studies have focused on isolated components of perception (e.g., face or motion processing), without addressing how these visual differences unfold within real-world, multisensory, dynamic environments [117,126]. Methodological discrepancies in eye-tracking paradigms and limited control for attentional factors make it difficult to disentangle primary neurobiological mechanisms from secondary adaptations to sensory overload or compensatory scanning styles [125,127,128].

Overall, the literature supported a consistent association between atypical visual processing and core behavioral features of ASD, particularly in social attention and perceptual organization. Behavioral and eye-tracking studies provided relatively robust evidence for altered visual strategies, especially under conditions of sensory uncertainty. In contrast, evidence based on neurobiological and neuroimaging findings remained heterogeneous and largely preliminary, due in part to small, developmentally diverse samples, and methodological variability. Many reported visual differences may therefore reflect alterations in sensory gain control or compensatory attentional strategies rather than uniform processing deficits. Larger, well-controlled, developmentally informed studies integrating objective instrumental measures with behavioral assessments and multisensory paradigms are needed to clarify causal mechanisms and establish clinical relevance.

### 4.3. Auditory Processing in ASD

Auditory alterations are frequently reported in individuals with ASD, encompassing both hyper-reactivity (hyperacusis) and hypo-reactivity, as well as abnormalities in auditory filtering, sound localization, temporal integration, and multisensory coordination [134]. Auditory hyper-reactivity has been associated with impaired attentional modulation and reduced habituation to auditory stimuli, manifesting as exaggerated reactions to everyday sounds that can lead to discomfort or even pain in response to stimuli typically well tolerated by neurotypical individuals [135]. Approximately 50–70% of individuals with ASD exhibit atypical behavioral responses to environmental sounds such as misophonia, phonophobia, or intolerance to loud environments, contributing to increased anxiety and avoidance behaviors [136]. These atypical responses have been shown to affect language processing, social communication, and learning, thereby reducing the ability to effectively process auditory information and playing a central role in defining the autistic phenotype [113,114].

In children with ASD, abnormalities in the auditory system have been identified, suggesting that these alterations may affect the ability to perceive and respond to auditory stimuli [137]. Consistently, neurophysiological studies have revealed atypical patterns of early auditory processing. Kujala et al. [138] examined the neural basis of aberrant speech and auditory processing in ASD and identified dysfunctions in the neural circuits responsible for language processing, as reflected by abnormal mismatch negativity responses—an event-related potential that indexes the brain’s automatic detection of unexpected changes in auditory input. These abnormalities are seen even in preverbal individuals, indicating a neurodevelopmental origin rather than learned behavior.

In different ASD rodent models, central auditory processing impairments emerged across the auditory pathway, from the brainstem to the cortex. Altered tonotopic maps, abnormal spectral and temporal resolution, and a consistent E–I imbalance associated with dysfunctional GABAergic signaling have been observed [139].

Similarly, neurophysiological studies in mouse models have shown increased N1 latency, reduced gamma-band coherence across auditory cortices, and exaggerated startle reflexes. These findings reflect impaired inhibitory interneuron function and abnormal thalamocortical connectivity [140]. Such models offer insight into the developmental trajectory of auditory dysfunction and are essential for evaluating therapeutic targets like GABA modulators.

Disruption in multimodal integration, especially between auditory and motor systems, has also been linked to mirror neuron system (MNS) dysfunction. Le Bel et al. [141] studied motor–auditory–visual integration and the role of the human MNS in communication disorders, highlighting how auditory sensory alterations may affect communication in individuals with ASD. The MNS, which integrates auditory, motor, and visual representations of action, is altered in ASD, potentially contributing to difficulties in language acquisition and imitation [141].

Another defining feature of auditory processing in ASD is atypical temporal integration, which affects the synchronization of auditory with other sensory streams, such as visual input. Stevenson et al. [81] found that individuals with ASD exhibit difficulties in aligning auditory and visual inputs, showing an enlarged TBW, particularly for audiovisual–speech. This temporal desynchronization may underlie speech perception difficulties in noisy environments or when lip-reading is required, as well as deficits in joint attention and social timing. These findings underscore that ASD is characterized not only by sensory hyper- or hypo-reactivity but also by temporal and integrative dysfunctions. Auditory performance also depends on the spectrotemporal complexity of the stimulus. Samson et al. [142] reported that individuals with ASD showed reduced accuracy and delayed responses when processing complex stimuli such as speech or musical chords. Conversely, they perform better than neurotypical controls on tasks involving simple tones or pitch detection [142].

This finding supports the theory of enhanced local but impaired global processing, which may account for the atypical decoding of auditory information in socially rich real-life contexts, filled with overlapping sounds. Individuals with ASD exhibit altered automatic grouping of sound features and reduced top-down modulation, meaning they may struggle to prioritize relevant auditory cues in noisy environments. This may contribute to auditory overload and difficulties with auditory attention shifting, which in turn affect classroom behavior, social interactions, and task execution [143].

Overall, behavioral and neurophysiological studies provided relatively robust evidence for altered auditory reactivity and temporal integration, especially under conditions of increased stimulus complexity or sensory load [81,138,142]. In contrast, evidence linking auditory alterations to specific neural pathways remained heterogeneous and often preliminary, largely due to small and clinically diverse samples and methodological variability [134,135,136,138]. Many auditory differences appear context-dependent and may reflect impaired sensory gating or compensatory attentional strategies rather than generalized perceptual deficits [134,135,136]. Larger, well-controlled, developmentally informed studies integrating behavioral, neurophysiological, and multisensory measures are needed to establish the developmental and clinical relevance of auditory alterations in ASD [81,138,142].

### 4.4. Olfactory and Gustatory Function in ASD

Olfaction and taste are fundamental sensory modalities for processing environmental stimuli and play a pivotal role in social cognition. Atypical processing of olfactory and gustatory stimuli has been identified as a typical feature of several neurodevelopmental disorders, including ASD [144]. Studies using the Sensory Profile questionnaire have revealed significant differences between individuals with typical development and those with ASD, while also highlighting substantial variability in the ASD group [145].

The sense of smell is a fundamental modality for socio-emotional processing [146]. It represents a system for detecting, identifying, recognizing, and storing a wide range of odors present in the environment [144]. Olfactory cues provide essential information for interacting with the environment, including assessing resources, opportunities, and potential threats, and they activate brain regions involved in emotional processing, attention regulation, and social stimulus interpretation, thereby guiding pro-social behavior [147,148]. Moreover, they also serve as a source of social information that shapes emotions and social behaviors, including sexual intimacy, aggression, and recognition [149]. Reports indicated that olfactory abilities are already present from infancy, functioning as an early warning system against harmful stimuli [144]. Through experience and learning, this system is refined, enabling the attribution of meaning to odors, improving discrimination, and enhancing attention to relevant stimuli [150].

Children with ASD often show poor hedonic discrimination of pleasant versus unpleasant odors, which impairs their ability to integrate effectively into social contexts [151]. The altered interaction between olfaction and emotion, described as “social dysosmia”, represents a potential mechanism underlying the emotional (and consequently social) processing deficits observed in individuals with ASD [152,153]. Additional alterations have also been observed in olfactory adaptation and habituation processes, leading to sensory and social processing difficulties, sometimes turning innocuous events, such as new olfactory experiences, into aversive ones [154,155,156]. This function reflects adaptive mechanisms of sensory acquisition and rejection, which regulate the amount of information received when a potential contaminant, threat, or positive chemical signal is detected [147].

Abnormal olfactory processing is closely linked to feeding difficulties, another hallmark of ASD. These are often characterized by food selectivity or food neophobia (fear of trying new foods) [157,158]. Parents frequently describe children with ASD as “picky eaters” [159]. Such behaviors may be associated with sensory processing alterations involving food consistency or temperature, or with the affective valence of olfactory food stimuli, which has been specifically correlated with food neophobia [151,160,161]. Indeed, children with ASD tend to prefer foods associated with a “familiar smell” [152]. A significant correlation has been documented between eating behavior in children with ASD and sensory processing, specifically oral sensitivity, which is associated with more severe eating impairments [162,163].

Two main sensory profiles have been identified in children with ASD. Hyper-reactivity children often display food refusal and dietary restriction; conversely, hypo-reactivity children may exhibit sensation-seeking behaviors, including binge eating, overeating, or oral self-stimulation [164,165].

Sensory hyper-reactivity or sensory defensiveness was first described by Ayres [166] in the tactile domain. It is now well established that tactile defensiveness correlates with feeding difficulties in ASD, especially oral hyper-reactivity [167]. This condition produces behaviors such as avoidance of certain food textures, discomfort with extreme food temperatures, and reluctance toward oral activities like tooth brushing [165]. For instance, children with ASD have been shown to consume significantly fewer fruits and vegetables compared to their neurotypical peers, leading to deficiencies in macronutrients, micronutrients, vitamins, and minerals [168]. Such food avoidance increases the risk of developing eating disorders, including avoidant/restrictive food intake disorder (ARFID) [169]. Feeding difficulties are often associated with behavioral problems, as children with ASD may be unable to verbalize their discomfort, resulting in disruptive manifestations (e.g., screaming or crying) during mealtimes. These behaviors not only exacerbate stress but also negatively impact family quality of life [170].

Behavioral evidence suggests that alterations in odor hedonic processing, habituation, and affective valuation may contribute to feeding difficulties and avoidance behaviors. However, despite growing interest in chemosensory processing in ASD, findings remained highly inconsistent across studies, due to substantial variability in sample characteristics—including age, cognitive profile, and clinical diagnosis of ASD versus elevated autistic traits—as well as by the use of heterogeneous assessment tools, ranging from subjective parent-report measures to psychophysical paradigms, which together limit comparability and generalizability across research efforts responses [144,151,163,164]. Moreover, the strong influence of environmental, familial, and cultural factors on food-related behaviors complicates the distinction between primary chemosensory alterations and learned avoidance or anxiety-driven responses [158,159,168,169,170].

As a result, evidence linking olfactory and gustatory atypicalities to core socio-emotional symptoms in ASD remains limited and is predominantly based on small, cross-sectional samples, preventing robust conclusions regarding developmental pathways and causality [144,152,153,154,164].

### 4.5. Interoception in ASD

Interoception refers to the ability to perceive and make sense of internal bodily signals, such as heartbeat, hunger, breathing, or visceral tension. This internal perception helps shape the embodied sense of self and plays a critical role in emotional awareness and social interaction. In ASD, however, this connection with internal states may be altered, potentially impacting emotional regulation, self-expression, and the recognition of physical and affective needs [101].

Interoceptive processing emerges from the integration of visceral afferents within a network of cortical and subcortical regions, including the insula, anterior cingulate cortex, and prefrontal systems. Although interoception is widely acknowledged as atypical in ASD, the degree and specific nature of impairment vary considerably, reflecting the heterogeneity of the condition. Nonetheless, group-level differences have been consistently documented, with a tendency toward reduced interoceptive awareness, or hypo-reactivity to internal cues, in individuals with ASD compared to their neurotypical peers [171]. To deepen our knowledge of how interoceptive differences contribute to behavioral and cognitive characteristics in ASD, a multidimensional research approach is needed, combining neuroimaging, psychophysiology, and self-report measures within unified theoretical frameworks. Supporting this direction, Yang et al. [172] examined interoceptive accuracy among children with ASD, children with co-occurring ASD and attention-deficit/hyperactivity disorder (ADHD), and typically developing children with high versus low autistic traits. Both ASD groups demonstrated significantly reduced interoceptive accuracy compared to their typically developing peers, while even neurotypical children with higher autistic traits performed more poorly than those with low autistic traits [172].

These findings highlighted that interoceptive differences in ASD and even in individuals showing elevated autistic traits may represent a fundamental dimension of neurodiversity with important implications for emotional processing and participation in daily life.

Overall, the literature suggested an association between altered interoceptive processing and core features of ASD, particularly in emotional awareness and self-regulation. Behavioral evidence pointed to reduced interoceptive awareness in individuals with ASD and, to a lesser extent, in those with elevated autistic traits. However, findings remained heterogeneous and strongly task-dependent, with discrepancies between subjective and objective measures. This variability was largely driven by small, predominantly high-functioning samples, reliance on cardiac-based paradigms, and inconsistent control of confounding factors such as anxiety and alexithymia.

## 5. Developmental Interactions Between Sensory Phenotypes and Comorbid Clinical Features in ASD

Sensory characteristics and their clinical consequences in individuals with ASD are not static across the lifespan and must be interpreted within the broader context of cognitive, communicative, and emotional development. Because most empirical research is based on verbally fluent and cognitively able individuals, the generalizability of existing sensory models is limited. In minimally verbal individuals and those with intellectual disability (ID), sensory difficulties often manifest primarily through observable behaviors—such as stereotypies, self-injury, or marked avoidance—especially in tactile and auditory domains. As standard assessment tools rely heavily on caregiver report, internal sensory states may be underdetected [173]. Evidence from cohorts including individuals with ID further showed that sensory profiles in ASD differ qualitatively from those in ID alone, with greater sensory sensitivity and avoidance contributing to heightened anxiety and behavioral dysregulation [173]. Recent findings in minimally verbal autistic children and adolescents additionally reveal heightened rates of atypical auditory behaviors (e.g., ear covering, humming), which are associated with receptive language difficulties and attenuated neural responses to sound-intensity changes, indicating modality-specific sensory dysfunction in this subgroup [174].

Across development, sensory characteristics do not evolve in isolation but interact with neurodevelopmental maturation and environmental demands. Sensory differences often emerge very early, frequently before age two, when children may show co-occurring patterns of hyper- and hypo-reactivity that influence attentional priorities, reduce social orienting, and shape emerging communication trajectories. As children grow, they frequently adopt avoidance or sensory-seeking behaviors as self-regulatory strategies [175]. In early childhood, sensory hyper-reactivity is common and strongly associated with internalizing symptoms, anxiety, and behavioral dysregulation. School environments often exacerbate these challenges: unpredictable routines, background noise, and multisensory demands may overwhelm sensory processing capacities, resulting in overload, escape behaviors, or reduced participation [176,177]. Neuroimaging findings parallel these behavioral patterns. Young children with ASD display heightened activation of sensorimotor, frontal, and cerebellar systems during sensory stimulation, supporting the idea of early neural hyper-reactivity [178]. Yet far fewer studies have characterized how these responses evolve through adolescence and adulthood. Adolescence represents a period of vulnerability: hormonal changes, increasing social expectations, and growing self-awareness can intensify sensory distress, even as some adolescents begin developing more effective coping strategies. Longitudinal and cross-sectional work suggests that developmental changes in the orbitofrontal and medial prefrontal cortices—regions involved in sensory integration and emotion regulation—may contribute to this variability [179]. In adulthood, findings become more complex. Some caregiver-based studies reported a reduction in the severity of hyper-reactivity with age, whereas self-report data indicated that sensory discomfort often remains prevalent, though manifesting in more internalized forms such as fatigue or cognitive overload rather than overt behaviors [180]. Consistent with this, Tavassoli et al. [19] have shown that adults with ASD frequently continue to experience significant sensory hyper-reactivity in daily life.

Sensory processes also interact dynamically with co-occurring conditions across development. Early sensory hyper-reactivity, hypo-reactivity, and sensory seeking can precede or amplify later emotional and behavioral difficulties [181].

Anxiety, in particular, showed a strong bidirectional association with sensory reactivity: unpredictable sensory input can trigger anticipatory worry and hypervigilance, while anxiety further lowers sensory thresholds, creating a self-reinforcing cycle of distress [182]. Sensory seeking and insistence on sameness may serve self-regulatory functions rather than merely reflecting repetitive behaviors [183]. Neuroimaging evidence implicated amygdala structure and connectivity differences in sensory-driven anxiety, and early difficulties in sound tolerance or olfactory processing might act as early markers for anxious trajectories [184].

During school-age years, co-occurring ADHD can complicate developmental interpretation of sensory behavior. Though sensory seeking may resemble hyperactivity, children with ASD and ADHD typically exhibit more profound sensory impulsivity and motor coordination challenges than those with ASD alone [185]. The partial overlap of sensory features across both conditions suggests shared neurobehavioral pathways in early development, which progressively differentiate as frontal–striatal networks mature [186].

Similarly, repetitive behaviors characteristic of ASD and Obsessive–Compulsive Disorder (OCD) show different developmental motivations. In ASD, repetitive actions may arise early as protective responses against overwhelming sensory input, while in OCD they emerge later as cognitive rituals driven by intrusive thoughts [186]. These distinctions appear to shift in adolescence, when increased self-awareness and social expectations may amplify the need for control and yield more internalized distress. Sensory integration difficulties and olfactory dysfunction represent additional links between ASD and OCD phenotypes, though research in primary OCD populations rarely considers sensory contributions [187].

Sleep disturbances, which are prevalent across all developmental stages, also reflect evolving sensory–regulatory interactions. Sensory hyper-reactivity may delay sleep onset in early childhood, while in adolescence reduced sleep duration and heightened physiological arousal can further amplify sensory sensitivities [188,189]. Although sensory-based interventions show preliminary promise for improving sleep, rigorous longitudinal studies remain scarce [190,191].

Across development, sensory challenges rarely disappear; rather, their expression transforms. Younger children often display overt avoidance or seeking behaviors, while adolescents and adults increasingly internalize sensory distress, drawing upon compensatory strategies and environmental control. Understanding these developmental dynamics is essential for identifying sensitive periods for intervention and tailoring supports that adapt to changing sensory, cognitive, and emotional needs over time.

## 6. Clinical Implications

Atypical sensory processing in ASD has broad clinical implications, influencing emotional, cognitive, and adaptive domains. Difficulties in sensory modulation interfere with self-care, feeding, and academic activities [192,193] and may hinder flexible behavior in novel or demanding environments [2].

Furthermore, cultural, social, and environmental contexts shape how sensory sensitivities are perceived and managed, as expectations regarding noise levels, crowding, physical touch, or feeding practices vary widely across communities and can either exacerbate or attenuate sensory challenges [194,195]. Sensory difficulties are more pronounced in classroom environments than at home, particularly for auditory processing, tactile sensitivity, social participation, and praxis. Excessive and unpredictable noise, crowding, frequent physical contact during group activities, rapid verbal instruction, and limited environmental predictability in school settings contribute to sensory overload and reduced functioning in students with ASD [196].

Among the three sensory response phenotypes, hyper-reactivity is most consistently associated with heightened anxiety, obsessive–compulsive traits, and emotional dysregulation, suggesting a bidirectional relationship in which increased sensory sensitivity amplifies anxiety and avoidance, thereby reinforcing hyper-reactivity itself [74,197]. This sensory phenotype also confers increased vulnerability to externalizing manifestations, including irritability, sleep disturbances, and behavioral dysregulation [197,198]. Within the family context, chronic sensory distress and behavioral rigidity often heighten caregiver burden and disrupt daily routines, underscoring the pervasive impact of atypical sensory processing on overall quality of life [199]. Additionally, hypo-reactivity and sensory seeking also contribute significantly to the clinical heterogeneity and impairment of ASD. Sensory hypo-reactivity has been associated with reduced responsivity to both social and nonsocial cues, which may hinder the development of reciprocal social communication [30]. Moreover, it has been linked to increased repetitive behaviors and reduced behavioral flexibility, suggesting broader effects on adaptive functioning [112]. Conversely, excessive sensory seeking has also been associated with impairments in attentional control, including difficulties in sustaining and shifting attention in response to environmental demands, which may further compromise social communication and adaptive engagement [31]. Collectively, these atypical sensory profiles contribute to the wide clinical heterogeneity of ASD and highlight the need for individualized assessment and tailored intervention strategies.

Understanding the individual sensory phenotype is therefore essential for accurate clinical assessment and intervention planning, as sensory processing is now recognized as a core feature of ASD closely related to attentional, motor, and affective regulation [20,200]. Evaluation of sensory processing differences typically combines proxy or self-report questionnaires (e.g., Sensory Profile), behavioral observation, and neurophysiological methods such as EEG or MEG [201]. Recent evidence showed that physiological markers provide an objective and clinically informative index of sensory reactivity in ASD. Children and adolescents with ASD exhibited stronger heart rate acceleration and reduced orienting responses to aversive sensory stimuli compared to typically developing peers, indicating altered autonomic regulation. Notably, physiological reactivity is most accurately predicted when parent-reported measures are combined with observational assessments of sensory behavior, supporting a multimodal evaluation framework. Together, these findings support the use of cardiovascular and electrodermal measures as complementary biomarkers of sensory reactivity in ASD [202].

Such heterogeneity further supports the conceptualization of distinct sensory profiles. In fact, recent studies have proposed multidimensional sensory profiles in individuals with ASD to inform and refine interventions targeting specific sensory domains and related behaviors. Using the Short Sensory Profile, which assesses sensory processing abilities across seven subscales, a five-cluster model of sensory phenotypes has been established [32]. The five clusters include sensory adaptive, generalized sensory difference, taste and smell sensitivity, under-responsive/sensory seeking and movement difficulties, and low energy/weak. These clusters are associated with distinct behavioral and adaptive profiles: individuals within each cluster share more similar sensory processing profiles than with individuals in other clusters [32] (see Table 2).

According to the five-cluster model, these sensory phenotypes also differ in their modality-specific profiles. Individuals with a sensory adaptive phenotype typically exhibit near-typical sensory processing across domains, whereas those with generalized sensory differences or taste–smell sensitivity often display atypical patterns of visual responsiveness. Likewise, under-responsive/sensory seeking and low-energy/weak profiles show distinctive multimodal sensory patterns—including differences in visual, auditory, and tactile processing—underscoring the heterogeneity and multidimensionality of sensory processing in ASD [32]. Understanding the sensory subtype of a newly diagnosed individual also contributes directly to personalized intervention planning. For example, it helps clarify whether the presenting problem behaviors are more likely to respond to a sensory-based desensitization protocol (as in taste/smell sensitive or generalized sensory difference profiles), an intervention targeting multisensory processing difficulties (as in generalized sensory difference profiles), or instead to non-sensory-based approaches (as in sensory adaptive profiles) [203].

## 7. Interventions Approaches

Although sensory processing differences in ASD are increasingly recognized, effective interventions remain unevenly implemented in clinical practice. From a cost–benefit perspective, many families are required to invest substantial time and financial resources in sensory-based therapies, while often encountering limited access to structured and evidence-informed services [195]. As a result, community-based providers and multidisciplinary teams play a central role in supporting individuals across developmental stages. However, current interventions often lack sufficient scientific validation, leaving clinicians and families with limited guidance regarding expected outcomes [195]. The following section outlines therapeutic approaches currently used to address sensory processing differences in ASD.

“Sensory interventions” represent a heterogeneous group of practices that differ in sensory targets, participant involvement, and settings, thereby limiting standardization and the comparability of outcomes across studies [204]. These interventions also operate through distinct mechanisms of change, an aspect that is essential for selecting the most appropriate approach. Such mechanisms include enhancing sensorimotor integration (e.g., Sensory Integration Therapy), reducing sensory distress (e.g., Sensory-based interventions), building tolerance through graded exposure (e.g., Sequential Oral Sensory Approach), engaging multisensory systems through structured physical activity programs, targeting emotional regulation (e.g., Cognitive Behavioral Therapy and mindfulness), and promoting neural plasticity through intensive sensory–motor stimulation [205,206,207,208,209,210,211].

Among these approaches, Sensory Integration Therapy (SIT), originally developed by Ayres [205], is one of the most widely implemented. It is a face-to-face intervention delivered by qualified occupational therapists who employ play-based sensory–motor activities, structured according to the just-right challenge. This approach aims to influence and modify the child’s sensory responses, thereby reducing distress and promoting improvements in motor skills, adaptive responses, concentration, and social interaction [9,212]. Despite its widespread clinical use, however, the empirical foundation of SIT remains limited. The highly individualized and therapist-dependent nature of SIT protocols constrains the ability to evaluate efficacy and generalize findings across populations. Future research should focus on developing standardized, empirically grounded protocols that integrate psychophysical and neurobiological assessments to strengthen methodological rigor and clinical applicability [14].

Alongside SIT, sensory-based interventions (SBIs) refer to adult-directed applications of specific sensory modalities that generally require minimal active engagement from the child and are designed to be seamlessly incorporated into daily routines [204]. According to the proposed mechanism, SBIs induce a temporary modulation of the individual’s physiological arousal state, resulting in decreased sympathetic activity and enhanced parasympathetic responses [206]. This autonomic shift is hypothesized to facilitate improved attention, behavioral regulation, and functional performance. Common examples include the use of weighted blankets, pressure vests, brushing protocols, and therapy balls. These interventions are usually performed in naturalistic settings by caregivers or educational staff, under the indirect supervision of occupational or physical therapists [204].

Effective interventions for feeding difficulties may especially benefit from a multidisciplinary approach. Occupational therapists can recommend sensory integration strategies, such as creating a calming environment (e.g., dimmed lights), to reduce overall arousal and facilitate coping with food-related sensory stimuli [165]. Dietitians may suggest alternative food presentations, avoiding aversive pairings or transforming fruits and vegetables into more palatable snacks [159,213]. Psychologists may apply behavioral interventions to progressively expand dietary repertoires and manage associated behaviors [214]. In this context, parental training under clinical supervision is essential for promoting appropriate food intake [215]. Play-based interventions, such as the Sequential Oral Sensory Approach (SOS), are desensitization protocols that proceed in six stages (visual tolerance, interaction, smell, touch, taste, and nutrition) and aim to gradually guide the child through exposure to and interaction with different foods [170,207,216,217]. Evidence shows that combining sensory, behavioral, and nutritional interventions with parent training effectively improves dietary intake and prevents severe nutritional deficiencies in children with ASD [218]. It is therefore essential to acknowledge individual variability in taste processing when planning interventions. Tailoring strategies to individual sensory profiles and nutritional needs is essential for optimizing outcomes [219].

More recently, structured exercise programs incorporating sensory integration principles—with targeted proprioceptive, vestibular, and tactile stimulation—have shown promise in supporting the developmental needs of children with ASD. Sensory-enriched physical activity, particularly when integrated into organized sports training, may enhance social responsiveness and emotional self-regulation. Multisensory sport-based activities, including obstacle courses, balance-oriented games, and guided team play, have been shown to facilitate sensory processing and foster peer engagement in everyday contexts [208].

Finally, complementary approaches, including Cognitive Behavioral Therapy, Mindfulness-Based Therapies, and parent training, have demonstrated effectiveness in reducing emotional dysregulation, anxiety, and caregiver burden associated with atypical sensory processing in ASD [197,209,210].

Expanding on traditional sensory-based and behavioral approaches, emerging neuroplasticity-based interventions, such as targeted sensory stimulation, neurofeedback, and non-invasive brain stimulation, aim to modulate neural connectivity and sensory integration at the cortical level, representing a promising avenue for future research and clinical application [211]. Overall, integrating sensory, behavioral, and neurobiological perspectives within multidisciplinary frameworks is essential to advance both the understanding and the personalized clinical management of sensory processing differences in ASD.

## 8. Future Directions and Conclusions

The evidence reviewed in this work confirms that in ASD sensory alterations are highly prevalent, clinically impactful, and closely related to social–communication difficulties, internalizing and externalizing symptoms, sleep disturbances, and repetitive behaviors. These findings provide a meaningful framework for interpreting the marked heterogeneity of autistic presentations and for understanding comorbid traits within the broader spectrum.

On the neurobiological level, a growing body of research has identified alterations in E–I balance, atypical connectivity patterns, and disruptions in predictive processing as key mechanisms contributing to sensory atypicalities in ASD. However, the identification of early and reliable biomarkers, such as habituation profiles and physiological indices of E–I regulation, remains an urgent priority to support earlier diagnosis and guide personalized intervention. Further advances are also expected from clarification of the link between genetic architecture and sensory phenotypes, as emerging genetic findings point to the possibility of increasingly specific and biologically informed subgroups. At the same time, research investigating immune-inflammatory contributions and neuromodulatory pathways, although promising, still requires stronger empirical validation in human samples. Translational studies targeting neuroplasticity and neuromodulation may provide innovative routes for enhancing sensory regulation and adaptive functioning.

**Figure 2:**
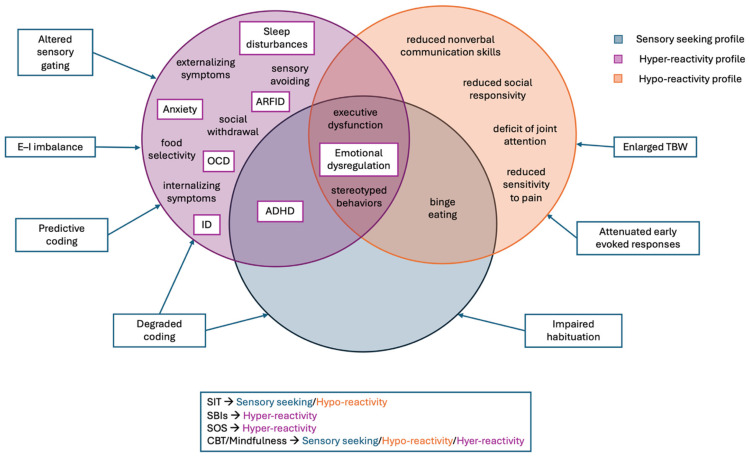
Conceptual integration of sensory phenotypes in autism spectrum disorder. The three core sensory profiles—hyper-reactivity, hypo-reactivity, and sensory seeking—are represented as partially overlapping domains. Behavioral manifestations are reported within each domain, while boxed elements indicate the most frequently reported comorbidities in individuals with ASD across sensory profiles. Owing to its more extensive representation in the literature, the hyper-reactivity profile is associated with a broader range of reported comorbidities. Putative neurobiological mechanisms related to sensory processing alterations are shown outside the sensory domains. The lower panel summarizes intervention strategies commonly used in relation to sensory-related behavioral features. Legend = ADHD: attention deficit/hyperactivity disorder; ARFID: avoidant/restrictive food intake disorder; ASD: Autism spectrum disorder; CBT: cognitive behavioral therapy; E–I: excitatory–inhibitory; ID: intellectual disability; OCD: obsessive–compulsive disorder; SBIs: sensory-based interventions; SIT: sensory integration therapy; SOS: Sequential Oral Sensory Approach; TBW: temporal binding windows.

In summary, sensory processing offers a powerful lens to understand ASD heterogeneity, and continued research across neural, behavioral, environmental, and lived-experience dimensions will improve both theory and clinical translation. Figure 2 schematically summarizes the reported associations between sensory phenotypes, clinical features, and underlying mechanisms discussed in this review.

However, important questions remain regarding the stability of these sensory profiles across development and their potential role in modulating treatment response [32]. Uncertainty also persists as to whether sensory differences cause comorbid conditions or result from them, and whether current findings are generalizable to individuals with ASD with lower verbal and cognitive functioning. Considering these uncertainties, intervention should prioritize environmental adaptation, sensory predictability, and alternative communication to reduce distress and support self-regulation, particularly in individuals with limited verbal ability.

Future research should adopt longitudinal and lifespan-oriented designs to clarify whether sensory phenotypes remain stable or evolve from childhood to adulthood, addressing the current gap in adolescent and adult populations [201,220]. Moreover, studies should investigate whether specific sensory configurations predict differential responsiveness to sensory-based, behavioral, or neuroplasticity-oriented interventions, thus advancing precision and personalization in ASD treatment.

Methodologically, existing research has largely relied on parent-report questionnaires, which, although useful for capturing everyday sensory experiences, are limited by subjectivity and recall bias. The adoption of harmonized and standardized sensory assessment tools will therefore be essential to improve external validity. Progress will therefore depend on the integration of more objective and multimodal approaches—including neuroimaging, electrophysiological, and behavioral measures—with innovative paradigms such as ecological momentary assessment, which enables real-time monitoring of sensory, emotional, and behavioral states in naturalistic contexts [27].

Overall, sensory processing provides a powerful framework through which autism’s heterogeneity can be understood from both mechanistic and clinical perspectives, offering concrete opportunities to improve diagnosis, prognostic stratification, and individualized intervention. By integrating neurobiological mechanisms with behavioral evidence and lived experience, future research can further refine mechanistic models and translate them into precision-based clinical strategies. A comprehensive, developmentally informed approach holds the potential to meaningfully enhance quality of life for individuals with ASD and their families. Continued advancement in this field will depend on clarifying causal pathways and ensuring broader inclusion across the spectrum, ultimately facilitating mechanism-based treatments and more accurate prediction of functional outcomes.

## Figures and Tables

**Table 1:** Core Sensory Constructs and Definitions: Definitions of sensory constructs are presented in a hierarchical structure, from broad concepts to specific neurobiological mechanisms.

Core Sensory Construct	Definition
**Sensory**	Active attempts to obtain sensory stimulation (e.g., touching surfaces repeatedly, rocking)
**Sensory phenotype**	The pattern of sensory reactivity and modulation that characterizes an individual with ASD
**Sensory profile**	The measurable configuration of sensory responses across modalities using standardized tools
**Sensory seeking**	The active pursuit of heightened or additional sensory input to achieve adequate sensory regulation
**Hyper-reactivity**	Exaggerated or aversive responses to sensory stimuli (e.g., covering ears to moderate noise)
**Hypo-reactivity**	Reduced or absent behavioral responses to sensory input (e.g., high pain threshold)
**Interoception**	Awareness and interpretation of internal bodily sensations (hunger, visceral pain, temperature, arousal)
**Multisensory integration**	The process by which sensory information from different modalities is combined to form a coherent percept; alterations in TBW have been reported in ASD and may contribute to atypical perception of social and environmental stimuli

Legend = ASD: Autism spectrum disorders; TBW: Temporal binding windows.

**Table 2:** The table summarizes the main behavioral and clinical characteristics associated with each sensory phenotype identified in the ASD population. Comparisons are based on *post hoc* analyses reported by Scheerer et al. [32], examining group differences across domains related to ASD, ADHD, and OCD traits, and motor/energy features. Higher scores generally indicate greater symptom severity or impairment, except for adaptive, communication, and social functioning, where higher scores reflect better performance. Overall, the five sensory phenotypes show distinct yet overlapping behavioral and clinical profiles. The SA group represents the most functional profile across all domains, characterized by better adaptive and social functioning and fewer behavioral difficulties. In contrast, the GSD phenotype shows the highest degree of impairment, marked by pervasive sensory alterations and elevated ASD and OCD traits. The TSS group exhibits an intermediate profile, with relatively adequate adaptive functioning but moderate ADHD-related features. Similarly, the URSS phenotype presents a mixed pattern, showing moderately reduced adaptive behavior and intermediate levels of ADHD-related features. Finally, the M/LEW group is characterized by motor coordination deficits, low energy, and elevated OCD features.

		SA	TSS	URSS	M/LEW	GSD
**ASD trait**	Adaptative behavior	↑↑↑	↑	↓	↓↓	↓↓↓
	Communication	↑↑↑	↑	↓	↓↓	↓↓↓
	Daily living	↑↑	↑↑	↓↓	↓↓↓	↓↓
	Socialization	↑↑↑	↑	↓	↓↓	↓↓↓
	Stereotypy	↓↓↓	↑↑	↑↑	↑	↑↑↑
	Repetitive behaviors	↓↓↓	↑	↑	↑↑	↑↑↑
	Self-injury	↓↓↓	↑	↑	↑	↑↑↑
**ADHD trait**	Hyperactivity	↓↓↓	↑	↑↑	↑↑	↑↑↑
	Inattention	↓↓↓	↓	↑↑	↑↑	↑↑↑
**OCD trait**		↓↓↓	↑	↓	↑↑	↑↑↑
**Motor Skills**		↑↑↑	↑	↑	↓↓↓	↓

Legend = ↓↓↓: significantly lower than all others; ↓↓: moderate lower; ↓: marginally lower; ↑↑↑: significantly higher than all others; ↑↑ moderate higher; ↑: marginally higher; ADHD: Attention Deficit Hyperactivity Disorder; ASD: Autism spectrum disorders; GSD: Generalized Sensory Differences; M/LEW: Movement Difficulties with Low Energy; OCD: Obsessive–Compulsive Disorder; SA: Sensory Adaptive; TSS: Taste and Smell Sensitivity; URSS: Under-Responsive and Sensory Seeking.

## Data Availability

No new data were created or analyzed in this study. Data sharing is not applicable to this article.

## Abbreviations

The following abbreviations are used in this manuscript:

ADHD	Attention Deficit Hyperactivity Disorder
ASD	Autism Spectrum Disorder
DSM-5	Diagnostic and Statistical Manual of Mental Disorders
EEG	Electroencephalography
E–I	Excitatory–Inhibitory
fMRI	Functional magnetic resonance imaging
GABA	Gamma-Aminobutyric Acid
MEG	Magnetoencephalography
MNS	Mirror neuron system
MRS	Magnetic resonance spectroscopy
OCD	Obsessive–Compulsive Disorder
OXT	Oxytocin
SBIs	Sensory-based interventions
SIT	Sensory Integration Therapy
SOS	Sequential Oral Sensory
TBW	Temporal binding window
TMS	Transcranial magnetic stimulation
